# Editorial

**Published:** 1873-03-01

**Authors:** 


### Medical Examiner.

*A Semi-Monthly Journal of Medical Sciences.*

EDITED BY

N. S. DAVIS, M. D., AND F. H. DAVIS, M. D.

Chicago, March 1st, 1873.

### EDITORIAL.

Summer Term of Medical Instruction.—The regular spring and summer course of instruction in the Chicago Medical College will commence on the first of April, and continue until the first of July. The arrangements for this course have been made more complete than for any previous year. It will embrace three regular didactic lectures per day, occupying from 9 to 12 o'clock A. M.; daily clinics in the Mercy Hospital and Davis Free Dispensary from 1½ to 3 o'clock P. M., the same as during the winter term; and instruction in Practical Chemistry and Microscopy in the Chemical and Microscopic Labratories.

All students who can remain in the city until the first of July will find this a most profitable course.

Material for dissection, which was deficient during a part of the winter, is now abundant, and will continue so during the spring. For further information, application can be made to Prof. D. T. Nelson, for printed circular.

---

The Sanitarian.—We have received an announcement stating that a new monthly journal, with the above title, will be commenced about the first of March, to be published by A. S. Barnes & Co., 111 and 113 Wilbro street, New York, and edited by A. N. Bell, M. D., of Brooklyn. It will be devoted to the cultivation of Sanitary Science and Improvement, as applicable in all the relations of life.

The Editor is admirably qualified for the management of such a work; and we trust the enterprise will be sustained by a liberal patronage.

It will be octavo size; forty-eight pages; monthly; at $3.00 per annum.

Commencement Exercises.—The public Commencement exercises of the Chicago Medical College, Medical Department of Northwestern University, will take place on Wednesday and Thursday, the 12th and 13th of March. The afternoon of Wednesday will be occupied in the reading of Theses and in the public examination of the candidates for graduation in class. The confering of the degrees, and the valedictory addresses, will be on the afternoon of Thursday.

---

Alumni Association of Chicago Medical College.—The annual reunion of this Society will be held in the College Hall, corner of Prairie avenue and Twenty-sixth street, on the 13th of March, at 10 o'clock A. M. The Secretary desires us to request every member to be present if possible. The public Commencement exercises of the College will be held in the afternoon of the same day.

---

American Medical Association.—The Twenty-fourth annual session of this Association will be held in St. Louis, Mo., commencing May 6th, 1873, at 11 A. M. We have just received a circular from the permanent Secretary, announcing the committees expected to report, etc., which we will publish in our next number.

---

Rush Medical College.—The Commencement exercises of this college took place in a public hall on Twenty-second street, on the evening of Wednesday, February 19th. The degree of M. D. was conferred by the President of the College, Prof. J. W. Freer, on 62 candidates; and the valedictory was delivered by Prof. Walter Hay, who was recently appointed adjunct Professor of Practical Medicine. A regular spring course of instruction is also to be given in connection with this College.

---

In Press.—A volume of Clinical Lectures, on Various Important Diseases, delivered in the Medical Wards of the Mercy Hospital, by Prof. N. S. Davis, collected and arranged by F. H. Davis M.D., will be ready for sale about the middle of the present month.

